# Desmoid tumour of the chest wall in paediatric post-operatory of heart transplant

**DOI:** 10.3389/fped.2022.860394

**Published:** 2022-11-28

**Authors:** Orival de Freitas Filho, Evelyn Sue Nakahira, Aurelino Fernandes Schmidt Junior, Estela Azeka, Marcelo Biscegli Jatene, Paulo Manuel Pego-Fernardes

**Affiliations:** ^1^ Department of Thoracic Surgery, Heart Institute (InCor) do Hospital das Clínicas, Faculdade de Medicina, Universidade de São Paulo, Brazil; ^2^ Department of Paediatric Heart Transplant, Heart Institute (InCor) do Hospital das Clínicas, Faculdade de Medicina, Universidade de São Paulo, Brazil

**Keywords:** paediatric heart transplantation, desmoid tumour, pacemaker, chest wall tumour, dilated cardiomyopathy (DCM)

## Abstract

We will report a case of a desmoid tumour (DT), which developed at the surgical site of the pacemaker after a late childhood heart transplant. Patients with idiopathic dilated cardiomyopathy followed up in the paediatric cardiology service. It evolved with the dissociation of ventricular rhythm caused by severe heart failure, which led to the implantation of a cardiac resynchronization device prior to heart transplantation. The progression to end-stage heart disease culminated in a heart transplant at 12 years old. One year after the transplant, at the age of 13 years, he presented a progressively growing mass on the generator site of the resynchronization device. The initial decision was to remove the device. During the removal surgery, there was no haematoma or fluid collection. However, there was a progression of the lesion. The lesion was biopsied with the anatomopathological diagnosis of a DT. Resection surgery happened 4 months after the start of the mass growth. At that time, the tumour reached 20 cm in diameter. The lesion infiltrated the pectoralis major muscle and this muscle was resected partially *en bloc* with the lesion. The defect had primary closure. The patient evolved without postoperative complications and was discharged on the 14th postoperative day. The surgical specimen came with negative circumferential margins. However, the deep margin was microscopically positive. Due to deep involvement, the patient underwent adjuvant radiotherapy. Currently, the patient is under clinical follow-up and has no evidence of tumour recurrence. DT is a rare tumour, with unpredictable courses. Surgery can be considered in the progression of lesions. Treatment is justified by long survival after a heart transplant and in DT patients. DT is a differential diagnosis to be considered in progressive growth lesions.

## Introduction

Heart transplantation is the treatment of choice for children with refractory congenital heart disease and cardiomyopathies. The tumours are transplant-related complications with challenging management.

A desmoid tumour (DT) is a rare type of tumour. DT consists of clonal fibroblastic proliferation from deep fascia or soft tissues. The tumour has unpredictable behaviour: spontaneous regression, maintenance, and growth are possible. DT does not give metastasis. However, local recurrence is frequent (1).

In this paper, we report DT in a heart transplantation patient. To our knowledge, it is the first case of such association. This case had challenging management due to the development and growth phase of adolescence in addition to the post-heart transplant status with the oncologic disease.

## Case report

A brown male had idiopathic dilated cardiomyopathy (DCM), which required a cardiac resynchronization device implant at 10-year-old due to severe heart failure with ventricular rhythm dissociation. He had no other past medical issues and also no familiar related disease. Due to the progression to end-stage heart disease, the patient had a heart transplant at 12 years old. The anatomopathological report of the explant confirmed idiopathic DCM. He used tacrolimus and mycophenolate for immunosuppression and without graft dysfunction. One year after the transplant, at the age of 13 years, he presented a progressively growing mass on the generator site of the resynchronization device. The initial decision was to remove the device. During the surgical procedure, there was no haematoma or fluid collection.

**Figure 1:**
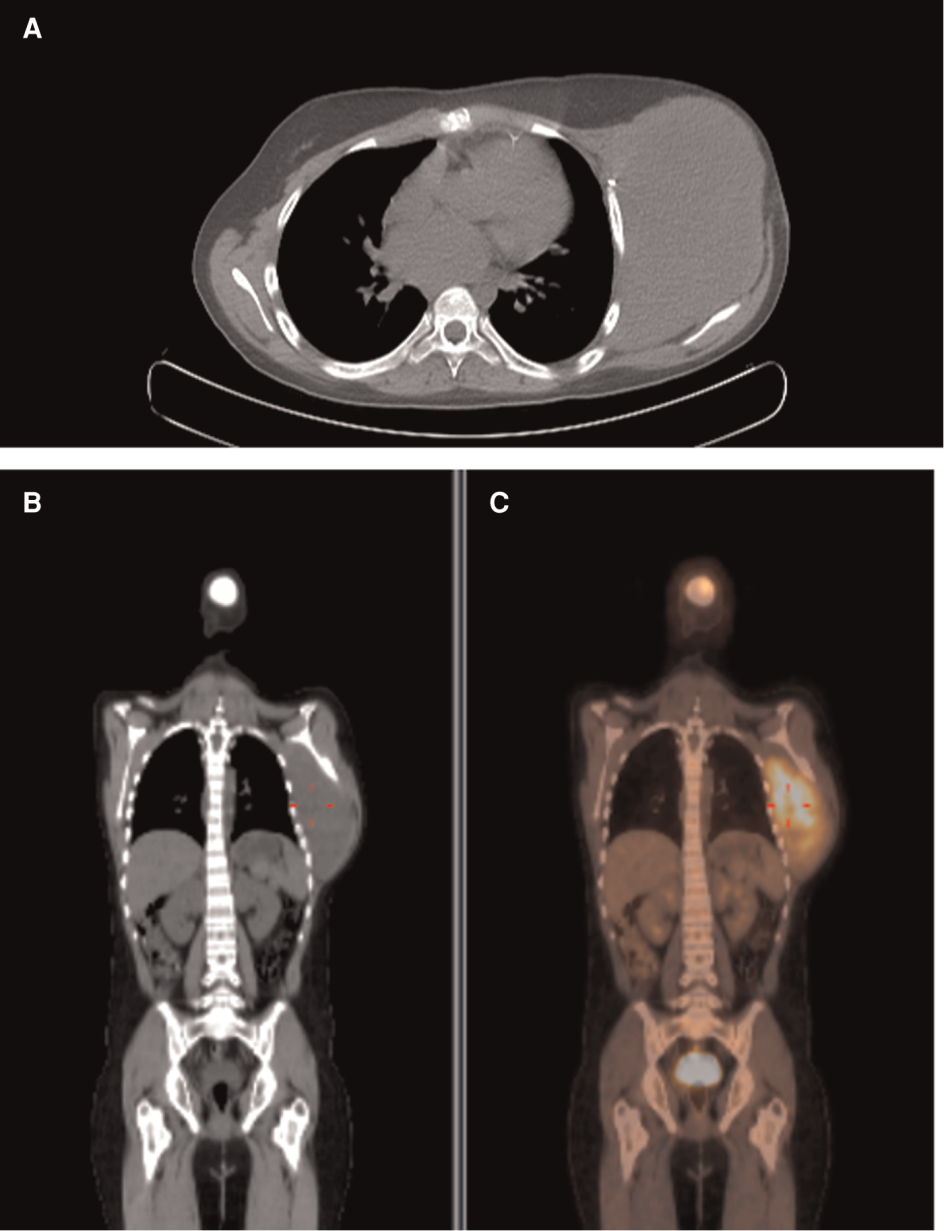
Preoperative PET image. Captant area in thoracic wall with SUVmax: 7.8. (**A**) Axial image. (**B**) Coronal image. (**C**) Coronal fusion image.

**Figure 2:**
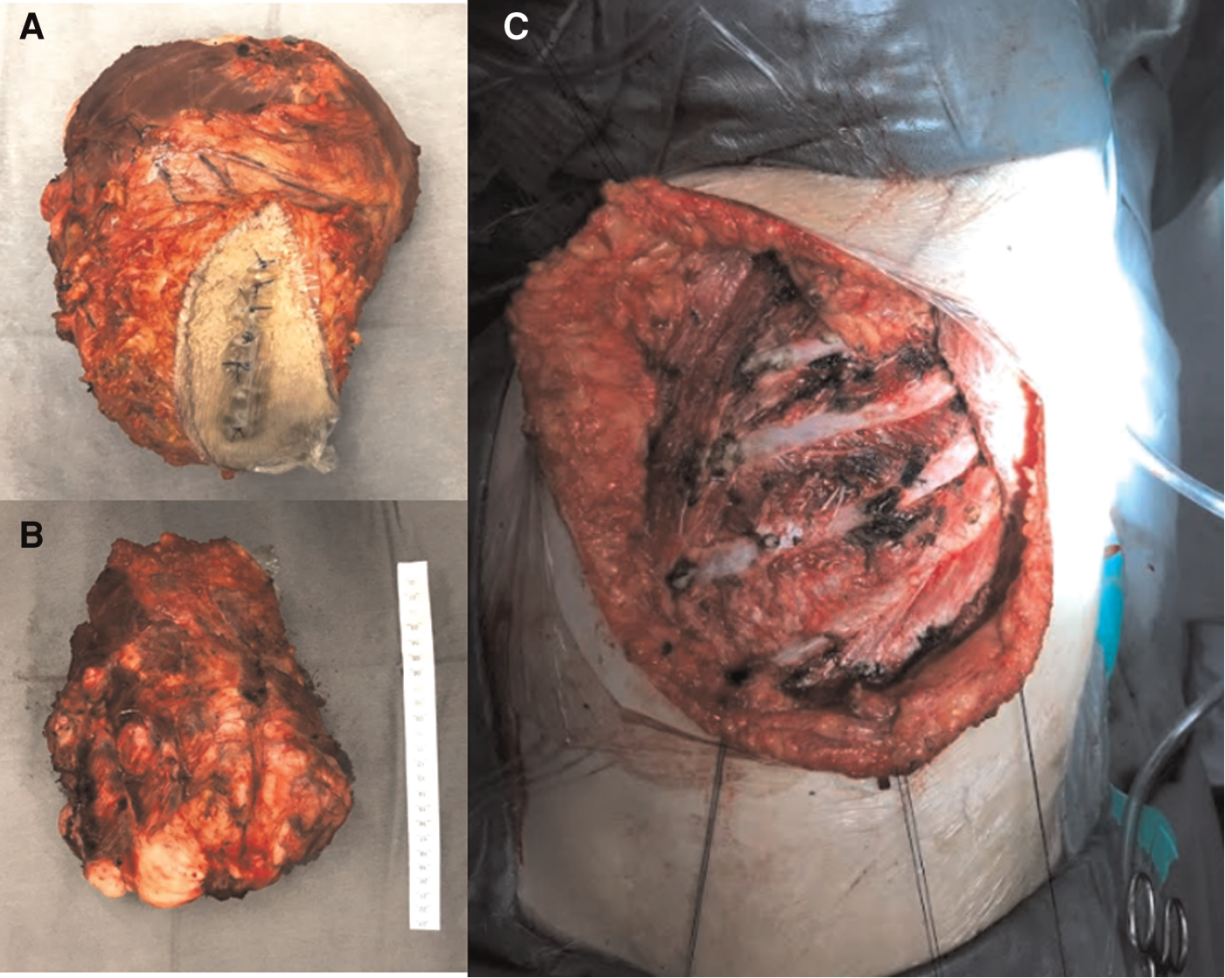
Intraoperative images. (**A**) Superficial side from the specimen. (**B**) Profound margem. (**C**) Resected area.

However, there was a progression of the lesion. A PET-CT was performed with the finding of an important uptake lesion at the thoracic wall with SUVmax 7.8. The lesion was biopsied with the anatomopathological diagnosis of a DT. Resection surgery happened 4 months after the start of the mass growth (Figure 1). At that time, the tumour reached 20 cm in diameter. The lesion infiltrated the pectoralis major muscle and microscopically did not invade the intercostal muscles or ribs. The pectoralis major partially was resected *en bloc* with the lesion (Figure 2). The defect had primary closure. The patient evolved without postoperative complications and was discharged on the 14th postoperative day.

**Figure 3:**
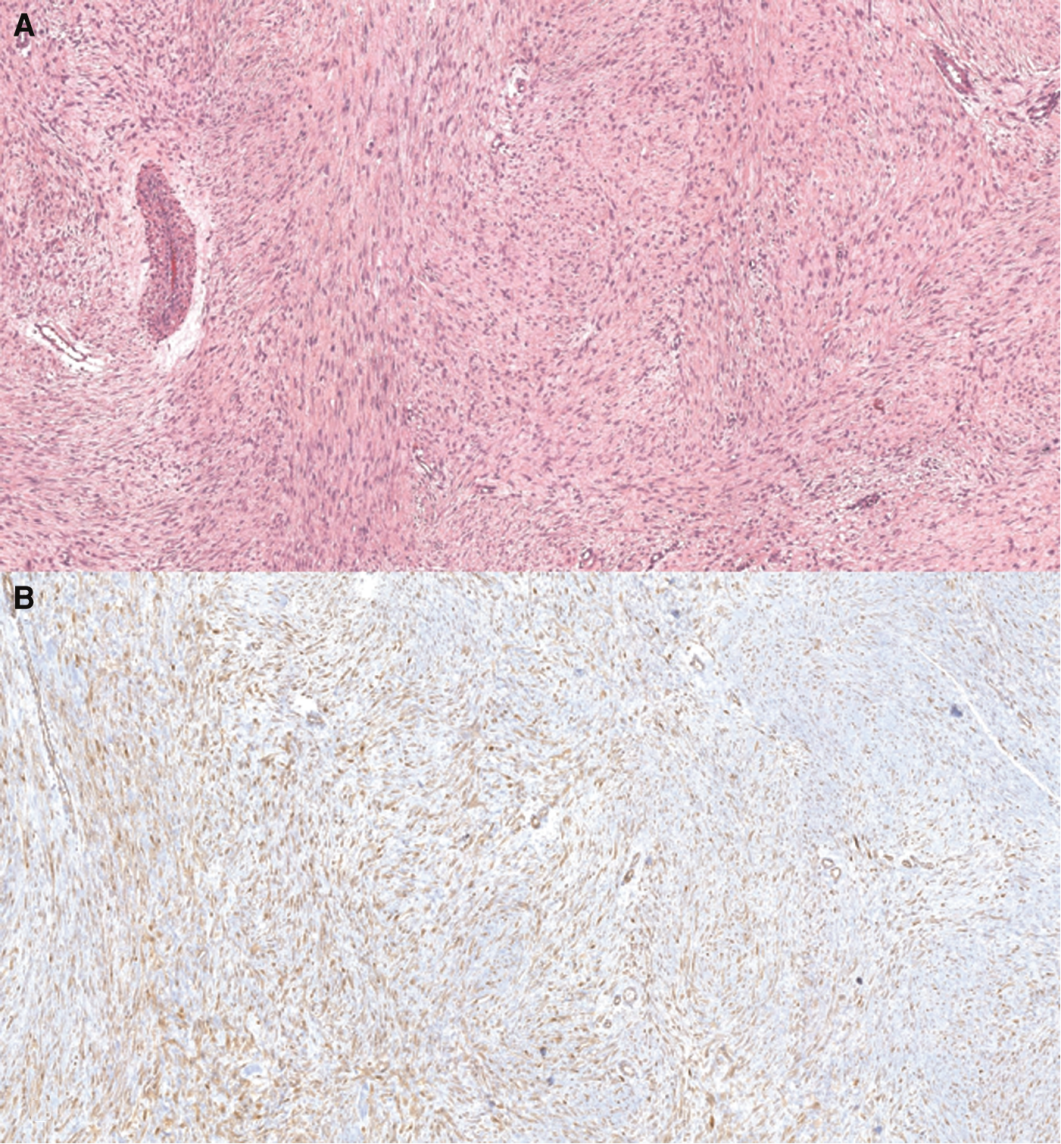
Pathological findings of the resected specimen. The tumour cells (fibroblasts and myofibroblasts) lack cytological atypia, form long, “sweeping” fascicles, and show nuclear beta-catenin expression. These findings are consistent with desmoid fibromatosis. (**A**) Hematoxylin-eosin, 100×. (**B**) Immunohistochemistry for beta-catenin, 100×.

The report from the surgical specimen confirmed that the lesion was a DT (Figure 3). The circumferential margins were negative. The profound margin was microscopically positive. Due to margin compromising, the patient received adjuvant 60 Gray radiotherapy administration 2 months after surgery. There is no evidence of tumour recurrence in a 4-year follow-up.

## Discussion

DT is associated with mutations in the Wnt/β-catenin pathway in the majority of cases. There is a hereditary type of disease with germline mutations in adenomatous polyposis coli (APC) being part of the familial adenomatous polyposis. There are also sporadic mutations related to DT (2). In the paediatric population, DT is associated with more gene mutations and a higher gene mutation rate than in adult cases, described in the literature the mutation rate of three exons of CTTNNB1 (3). DT can be related to local trauma, and surgery is associated with 28% of DT cases (4). In this case report, DT appeared at the surgical site of a resynchronization device implant, which is a previous site of surgical trauma.

In one study with 192 included patients with DT, 11% of the sample was paediatric. In this subgroup, the median age is 15 years, with female predominance of 64%. The median tumour size was 5.3 cm. In children, all tumour sites were extra-abdominal. Lesions were predominant in the extremities, and only 15% were in the trunk (2).

There is no report of DT after a heart transplant in the literature. There are reports of DT in patients with lung, liver, and renal recipients (5). Transplant patients are at higher risk for developing tumours due to immunosuppressive therapy, particularly non-melanoma skin cancer and non-Hodgkin's lymphoma (6). In the literature, 12.1% of heart post-transplant patients had malignancies diagnosed, and in this population, post-transplant lymphoproliferative diseases were the most common (7).

Once a growing mass develops, it should trigger an investigation. DT is diagnosed by an anatomopathological study of the lesion. Active surveillance is the initial approach to DT for asymptomatic patients (1). The unpredictable course of DT can justify this. Moreover, there is no difference in event-free survival between active surveillance and surgery (1). If there is a growing lesion, active treatment (with medical treatment and/or surgery) should be considered (1).

There are some considerations for surgery management. Between R0 and R1, there is no difference in the recurrence outcome. R1 resection is acceptable when the functionality is an issue (1). Based on the same outcome from R0 and R1 surgery, in the paediatric populations that are still at a time of physiological growth, sparing of structures should be considered and the decision is performed after multidisciplinary discussion.

Radiotherapy seems an adjuvant approach for DT, especially after R1 resection. However, the difference between surgery and surgery plus radiotherapy is not statistically significant (1).

DT has a high recurrence rate (24%–76%), despite the 10-year overall survival rate being higher than 90% (8). In paediatric heart transplants, post-operatory survival is 13.1 years for those more than 11 years of age at transplant (9). Long-term survival after transplant or DT justifies intervention in this case.

New lesions after transplantation require investigation and diagnosis, considering immunosuppression. DT is a rare aetiology; however, this disease consists of differential diagnoses for growing lesions. Regarding the heart transplant, there is a good outcome in post-operatory survival, which justifies the treatment of DT. Paediatric patients also have the particularity of developing tissues, and this should be taken into account to decide the therapeutic approach.

## Data Availability

The raw data supporting the conclusions of this article will be made available by the authors, without undue reservation.
